# Development and Validation of a Novel Diagnostic Model for Childhood Autism Spectrum Disorder Based on Ferroptosis-Related Genes

**DOI:** 10.3389/fpsyt.2022.886055

**Published:** 2022-05-12

**Authors:** Xiaolu Wu, Ran Li, Qin Hong, Xia Chi

**Affiliations:** ^1^Department of Child Health Care, Nanjing Maternity and Child Health Care Hospital, Women’s Hospital of Nanjing Medical University, Nanjing, China; ^2^Shanghai Institute of Hematology, State Key Laboratory of Medical Genomics, National Research Center for Translational Medicine at Shanghai, Ruijin Hospital, Shanghai Jiao Tong University School of Medicine, Shanghai, China

**Keywords:** autism spectrum disorder, diagnosis, ferroptosis, random forest, piperaquine

## Abstract

Autism spectrum disorder (ASD) is a highly genetic heterogeneous neurodevelopmental disorder, which is usually considered a heritable and heterogeneous neurodevelopmental disorder and has caused a great burden to society and families. Emerging roles of ferroptosis have been observed in neurological disorders. This study aimed to construct a diagnostic model based on ferroptosis-related genes (FRGs) to contribute to the early and precise diagnosis of childhood ASD. In the candidate FRGs, we identified 27 differentially expressed genes (DEGs) between ASD patients and typically developing (TD) controls. Four key FRGs were identified using the random forest analysis for further analysis. Utilization of the four gene expression, we constructed a diagnostic model and the AUC value in the training dataset (GSE18123) is 0.7002. We deem that a patient with a score less than 0.9904 is likely to have ASD. Three validation datasets (GSE111176, GSE113834, and GSE28521) were collected and the AUC value is 0.7442, 0.7444, and 0.6474, respectively. A multi-factor regulatory network based on four FRGs indicated that RORA, EAF1, NFYB, miR-4703-3p, and miR-6073 may play a role in the development of ASD. In addition, we found piperaquine may have the potential to be a promising drug for the treatment of ASD. Overall, we constructed a diagnostic model of childhood ASD, which could contribute to the precision diagnosis and timely treatment of childhood ASD.

## Introduction

Autism spectrum disorder (ASD) is a set of heterogeneous genetically complex neurodevelopmental disorders, characterized by a deficit in social communication and interaction, and lasting impairments in restricted, stereotyped, and repetitive patterns of behavior or interests (1). The prevalence of ASD continues to increase worldwide since the first epidemiological study, latest study reported an incidence of 1 in 54 children worldwide (2). Males are four times more likely to develop autism than girls (3). Several complex underlying mechanisms were involved in the development of ASD, such as genetic and epigenetic effects, inflammation, oxidative stress, neurotrophic factors, and hypoxic damage (4–6).

Early diagnosis and appropriate intervention for childhood ASD can improve ASD outcomes. However, diagnosis of autism was often delayed until mid-childhood because of complex symptoms, insufficient screening practices, the lack of clinical access, low sensitivity of autism screening instruments, and ignorance of early warning signs (7, 8). In addition, all present medications that have evidence to be beneficial to autism are aimed at related symptoms, rather than directly at autism symptoms (including repetitive behaviors or social communication) (9). With the development of sequencing technologies, abundant transcriptomic analyses improve our understanding of ASD and allow us to develop a novel model contributing to the early precise diagnosis of childhood ASD.

In recent years, ferroptosis is a newly identified iron-dependent form of programmed cell death, primarily caused by the imbalance of oxidation and anti-oxidation in the body (10). Ferroptosis is typified by lipid peroxidation and depends upon the severe lipid peroxidation of intracellular iron accumulation and generation of reactive oxygen species (ROS) (11). A growing body of evidence shows that ferroptosis is strongly implicated in a variety of diseases, including cancers, cardiovascular diseases, kidney damage, and nervous system diseases (12). Ferroptosis is closely linked to the occurrence and development of neurodegenerative diseases, strokes, and brain tumors. It is also involved in the development, maturation, and aging of the nervous system (13, 14). Many studies have found a link between ASD and elevated oxidative stress and ASD is contributed to by oxidative stress in several ways, including protein post-translational changes, abnormal metabolism (e.g., lipid peroxidation), and toxic buildup (e.g., ROS) (15). However, the definite role of ferroptosis in ASD is still unclear. In this study, we aimed to explore the potential association between ferroptosis-related genes (FRGs) and ASD and hope to develop a novel diagnostic model to contribute to the early and precise diagnosis of childhood ASD.

## Materials and Methods

### Data Acquisition

The transcriptome data and clinical information of ASD and typically developing (TD) samples were downloaded from GSE18123 and GSE111176 datasets. The external validation data were downloaded from GSE113834 and GSE28521. The characteristics of the four datasets are detailed in Supplementary Table 1. Briefly, the sample size of normal control is 82, 126, 12, and 40, respectively (from left to right, GSE18123, GSE111176, GSE113834, and GSE28521); the sample size of ASD is 41, 119, 15, and 39, respectively (from left to right, GSE18123, GSE111176, GSE113834, and GSE28521). Tissues are sourced from blood (GSE18123 and GSE111176) or the brain (GSE113834, and GSE28521). The information on age, gender, time of collection of blood samples, and postmortem parameters was incomplete. The downloaded data were normalized by log2-transformed [log2(*x* + 1)]. All samples included in this study were accorded with the inclusive criteria: (1) Homo sapiens; (2) ASD and TD samples; (3) Availability of transcriptome data; and (4) Validation data including the expression of key genes included in the model. Three hundred thirty-five FRGs were obtained from the FerrDb database^1^. The rest FRGs were acquired from three published articles (16–18). After integrating these genes, we at last acquired 376 FRGs for further analysis.

### Identification of Differentially Expressed FRGs

The “limma” package from R software was used to identify DEGs between ASD and TD groups. Only genes with *P* < 0.05 were considered as DEFRGs.

### Functional Enrichment Analysis

The “clusterProfiler” R package was used to conduct Gene Ontology (GO) and Kyoto Encyclopedia of Genes and Genomes (KEGG) analyses.

### Establishment of a Ferroptosis-Related Diagnostic Model

After obtaining 27 differentially expressed FRGs, random forest analysis was performed to further identify key FRGs. The “RandomForest” R package was used to conduct random forest analysis. Parameters were set to default and genes with Gini values greater than 1 were considered key genes. After taking the intersection of GSE18123 and GSE111176, we obtained four key FRGs. Multiple logistic regression was applied to construct a ferroptosis-related diagnostic model. The diagnostic score was calculated using the equation: Score = AKR1C3 expression*(−0.2837) + CEBPG expression*(−0.7620) + DDIT4 expression*(−0.5943) + LAMP2 expression*(−0.9101) + 21.38.

### Evaluation of the Ferroptosis-Related Diagnostic Model

Every individual from the Gene Expression Omnibus (GEO) database was allocated a diagnostic score derived from FRDM. Model specificity and sensitivity were assessed by calculating the area under the curve (AUC) values of ROC curves. The training cohort was GSE18123; the internal validation cohort was GSE111176; the external validation cohort was GSE113834 and GSE28521.

### Regulatory Network

The microRNA (miRNA), long non-coding RNA (lncRNA), and transcription factor (TF) that interact with key genes were extracted from microRNA Data Integration Portal (mirDIP), starBase, and transcriptional regulatory relationships unraveled by sentence-based text mining (TRRUST) databases, respectively. Cytoscape software was used to display the multi-factor interaction network of key genes.

### Molecular Docking

The protein structure information was downloaded from the PDBbind database^2^. Compounds’ structure information was downloaded from DrugBank^3^. AutoDock Vina was used to perform molecular docking, and compounds were filtered according to the binding energy. Screening parameters were set as Affinity < −7. PyMOL software was used to plot a protein-compound binding diagram.

### Statistical Analysis

The R software (version 4.1.1)^4^ was used to perform all statistical analyses. Student *t*-test or one-way ANOVA was applied to assess differences between groups. *P* < 0.05 was considered statistically significant.

## Results

### Identification of Differentially Expressed FRGs and Enrichment Analysis

**FIGURE 1:**
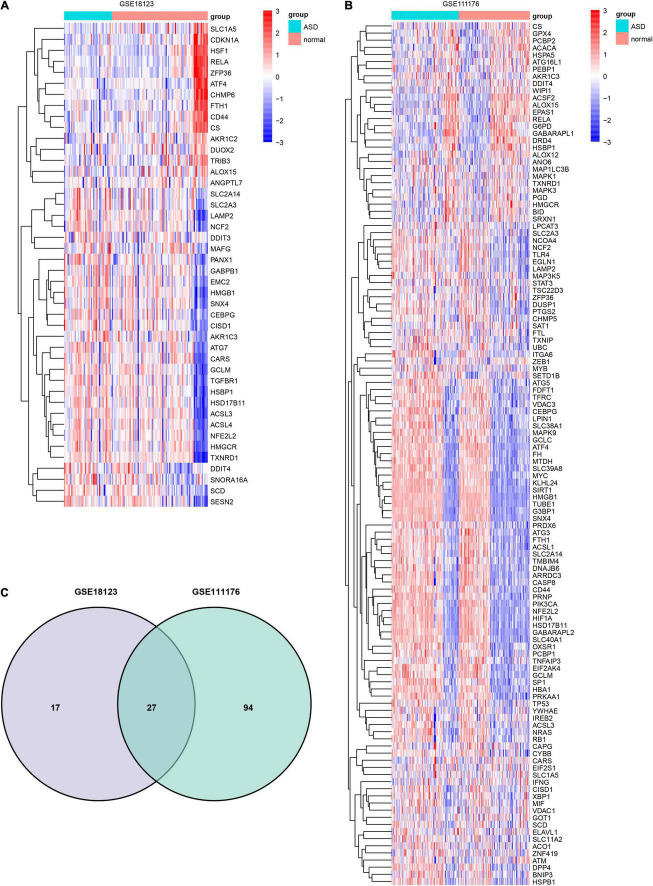
Identification of DEFRGs. Heatmap of DEFRGs from GSE18123 **(A)** and GSE111176 **(B)**. **(C)** Venn diagram for DEFRGs.

**FIGURE 2:**
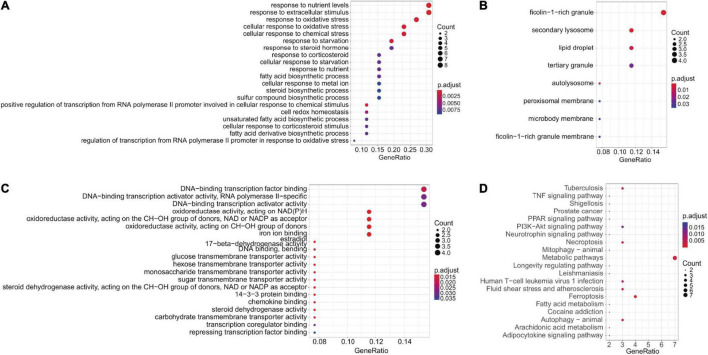
Enrichment analysis. **(A–C)** GO enrichment analysis (BP, CC, and MF, respectively). **(D)** KEGG enrichment analysis.

Forty four FRGs were extracted from the GSE18123 dataset (Figure 1A). One hundred twenty-one FRGs were extracted from the GSE111176 dataset (Figure 1B). After taking the intersection of FRGs from two datasets, we at last obtained 27 FRGs for further analysis (Figure 1C). The top 20 terms of the 27 FRGs’ enrichment analysis were displayed in four parts, including biological process (Figure 2A), cell component (Figure 2B), molecular function (Figure 2C), and KEGG (Figure 2D).

### Random Forest Analysis

**FIGURE 3:**
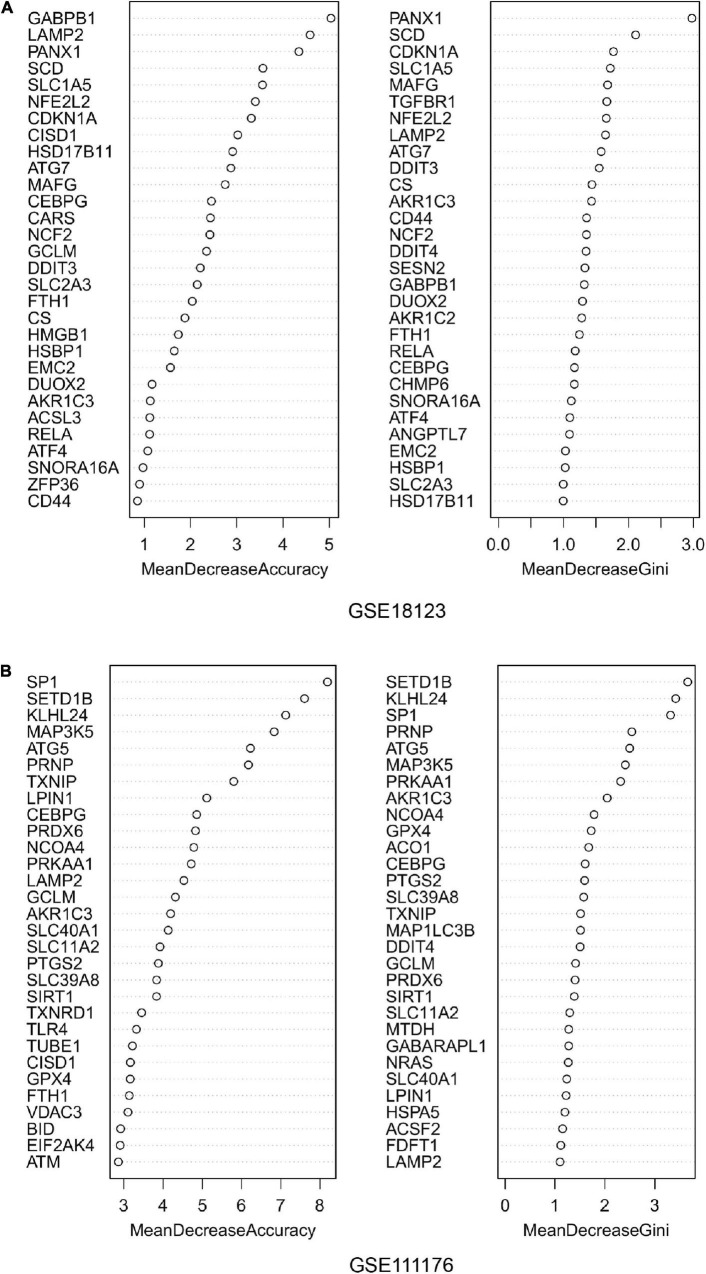
Random forest analysis. **(A)** Accuracy value and Gini value of candidate ferroptosis-related genes in GSE18123. **(B)** Accuracy and Gini values of candidate ferroptosis-related genes in GSE111176.

For the differentially expressed FRGs, random forest analysis was performed to further identify key FRGs. The top 30 genes with high accuracy and Gini value were displayed in Figures 3A,B. Genes with Gini value >1 were selected and we obtained 28 genes in GSE18123 and 35 genes in GSE111176. Integrating the key genes from two datasets, we at last acquired 4 key FRGs (Supplementary Figure 1).

### Expression of Ferroptosis-Related Genes in GSE18123 and GSE111176

**FIGURE 4:**
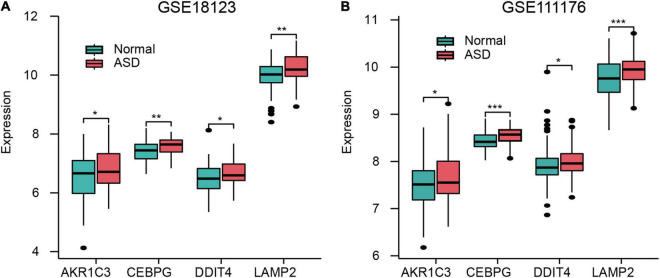
Expression of FRGs in GSE18123 **(A)** and GSE111176 **(B)**. *p < 0.05, **p < 0.01, ***p < 0.001.

AKR1C3, CEBPG, DDIT4, and LAMP2 were all highly expressed in ASD samples in both GSE18123 (Figure 4A) and GSE111176 datasets (Figure 4B). Additionally, in GSE18123, FRGs have positive correlations except for DDIT4 and LAMP2 genes (Supplementary Figure 2A). In GSE111176, AKR1C3 and DDIT4 have a positive correlation. CEBPG was negatively associated with DDIT4; while positively associated with LAMP2. DDIT4 and LAMP2 have a negative correlation (Supplementary Figure 2B).

### Construction and Evaluation of a Ferroptosis-Related Diagnostic Model

**FIGURE 5:**
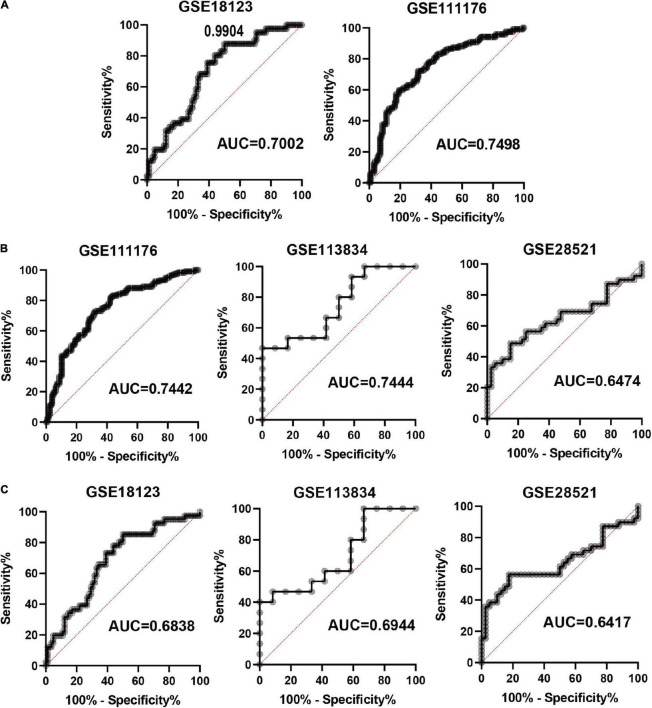
Evaluation of the constructed diagnostic model. **(A)** ROC curves in development datasets. **(B)** ROC curves in validation datasets based on the model from GSE18123. **(C)** ROC curves in validation datasets based on the model from GSE111176.

Based on the four FRGs, we constructed diagnostic models using multiple logistic regression in GSE18123 and GSE111176, respectively. Then, ROC curves were used to evaluate the model specificity and sensitivity. The AUC value for the diagnostic model in GSE18123 is 0.7002; in GSE111176 is 0.7498 (Figure 5A). To further evaluate the reliability of the two models, external datasets were used to perform validations. For the model based on GSE18123, validation datasets including GSE111176, GSE113834, and GSE28521 were adopted and the AUC value is 0.7442, 0.7444, and 0.6474, respectively (Figure 5B). For the model based on GSE111176, validation datasets including GSE18123, GSE113834, and GSE28521 were adopted and the AUC value is 0.6838, 0.6944, and 0.6417, respectively (Figure 5C). Hence, the diagnostic model based on GSE18123 has a better diagnostic performance. The cutoff value of the diagnostic model was determined to be 0.9904 based on the Youden index (19); a diagnostic index ≤0.9904 indicated an ASD, and a diagnostic index >0.4 indicated its absence.

### Multi-Factor Regulatory Network

**FIGURE 6:**
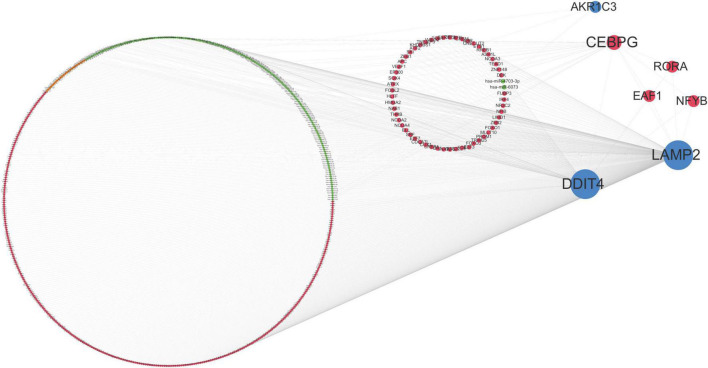
Multi-factor regulatory network. Orange dots represent lncRNAs, red dots represent transcription factors (TF), green dots represent miRNAs, blue dots represent mRNAs, and the size of dots represents the number of regulatory genes.

According to the interaction degree, RORA, EAF1, and NFYB are the only three transcription factors that can interact with CEBPG, DDIT4, and LAMP2. In addition, hsa-miR-4703-3p can regulate LAMP2 and AKR1C3; hsa-miR-6073 can regulate DDIT4 and LAMP2 (Figure 6). These results indicated that TFs and miRNAs may play an important role in the development of ASD.

### Potential Therapeutic Compounds

**FIGURE 7:**
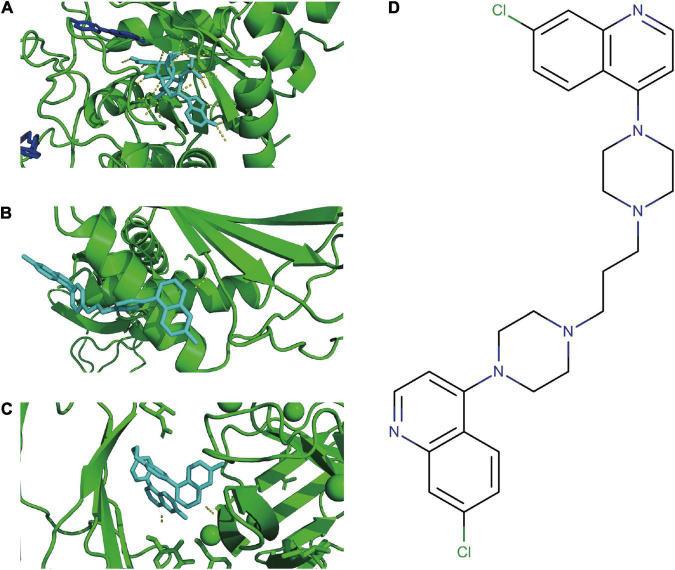
Molecular docking. Docking conformation of DB13941 with AKR1C3 **(A)**, DDIT4 **(B)**, LAMP2 **(C)**. The compound structure of DB13941 **(D)**.

According to the PDBbind database, AKR1C3, LAMP2, and DDIT4 have available spatial structure information for subsequent analysis. We found only piperaquine could dock to all three FRGs with affinity < −7. The docking conformation of piperaquine to FRGs was displayed in Figure 7A (AKR1C3), Figure 7B (DDIT4), and Figure 7C (LAMP2). Figure 7D showed the compound structure of piperaquine. The docking score was shown in Supplementary Table 2.

## Discussion

At present, the ASD diagnosis was confirmed using clinical expert assessment including the Autism Diagnostic Interview-Revised (ADI-R) and the Autism Diagnostic Observation Schedule (ADOS), but only a limited number have been rigorously tested for diagnostic accuracy (20). In this study, based on transcriptome data, we constructed a ferroptosis-related diagnostic model to distinguish ASD patients from the TD population. We used three validation datasets to evaluate the precision of this model and the AUC value is 0.7442, 0.7444, and 0.6474, respectively. Hence, this diagnostic model may have the potential to be applied in the future.

To date, the four genes in this study have not been reported in ASD. CCAAT Enhancer Binding Protein Gamma (CEBPG) is a member of the C/EBP family and has the sequences required for DNA binding and heterodimer formation, but lacks the sequences required for transactivation (21). CEBPG appears as a stress-induced gene in some genome-wide expression studies, which can stimulate promoters of IL-6 and IL-8 in B cell lines (22). Previous studies have implicated CEBPG as an antioxidant regulator that controls redox homeostasis in normal and cancer cells (23). The neurobiology of ASD is considered related to oxidative stress (24) which indicated that CEBPG may be involved in the development of ASD. Aldo-Keto Reductase Family 1 Member C3 (AKR1C3) is known as a hydroxysteroid dehydrogenase, which exists in many normal human tissues at varying levels (25). The mRNA expression and enzyme activity of AKR1C3 in subcortical white matter were significantly higher than in the cerebral cortex, and AKR1C3 activity in adults was higher than in children, but no gender differences were observed (26). In addition, the antioxidant response element (ARE) could bind to the AKR1C3 promoter indicating that oxidative and electrophilic stress might regulate AKR1C3 expression (27). Due to the upregulation of AKR1C3 being closely correlated with various diseases, a large range of research on AKR1C3 inhibitors has been executed in the past few years, which could have the potential to be applied to ASD patients. Lysosomal Associated Membrane Protein 2 (LAMP2) is a major lysosomal membrane glycoprotein, which plays a role in multiple biological processes including antigen presentation, oxidative stress, and regulation of T lymphocyte responses (28). LAMP2 is ubiquitously expressed in the central nervous system and has been reported involved in the development of Parkinson’s disease and Alzheimer’s disease (29, 30). DNA Damage Inducible Transcript 4 (DDIT4), is known as a HIF1A responsive protein that promotes oxidative stress-dependent cell death (31). In addition, numerous studies have suggested that DDIT4 was crucial for optimal T cell proliferation and survival (32). Abnormal immune system regulation is involved in the pathophysiological process of ASD, including T cell-related signaling pathways (33). Overall, all these four genes are associated with oxidative stress or immune response, which provides clues for the exploration of the pathogenesis of ASD.

To elucidate the potential mechanism of the four genes in ASD, we conducted a multi-factor regulation network. We identified that RORA, EAF1, NFYB, miR-4703-3p, and miR-6073 might play a role in the development of ASD. RAR-Related Orphan Receptor A (RORA) is a ligand-dependent nuclear receptor that regulates gene transcription. Recently, studies have identified RORA as a novel candidate gene for ASD, which may be conducive to the known pathophysiology, behaviors, and sex bias of ASD (34). ELL Associated Factor 1 (EAF1) is one of the EAF family members, which plays an essential role in tumor suppression and embryogenesis (35). EAF1 and EAF2 can suppress Wnt/β-catenin signaling to affect neuroectodermal and mesodermal patterning (36). Nuclear Transcription Factor Y Subunit Beta (NFYB) plays a fundamental role in proliferation by binding to and regulating the transcription of numerous cell cycle regulatory genes (37). So far, there have been no reports on miR-4703-3p and miR-6073, and our study provides insights into the potential role of these miRNAs in ASD.

The classical therapeutic drugs for many diseases may have unexpected curative effects in other diseases, which is an approach known as drug repurposing. Piperaquine is a bisquinoline antimalarial drug, which is known as an effective drug to treat malaria, similar to chloroquine (38). We found that piperaquine has good docking scores with all three FRGs, which indicated piperaquine could be a promising drug for the treatment of ASD. The immune complex consists of brain tissue antigens and nerve-specific autoantibodies that can penetrate the blood-brain barrier and damage nerve tissue of children, resulting in cognitive, language development, and social communication disorders of children (39). Previous studies have shown that chloroquine has anti-inflammatory and antiviral effects, which are used to treat many diseases such as systemic lupus erythematosus (SLE), antiphospholipid antibody syndrome (APS), Middle-east respiratory syndrome, and HIV infection (40–42). Virus infection can cause irreversible damage to the central nervous system which produces ASD-related symptoms (43). Hence, piperaquine may play a role in the treatment of ASD through the immune-related pathway. In the future, studies need to be performed to explore the potential effect of piperaquine on ASD patients.

To a certain extent, several limitations of this study should not be ignored. (a) Clinical information was limited due to the retrospective data from the GEO database; (b) the number of samples in this study was relatively small, especially in the GSE113834 and GSE28521. In addition, the data from GSE113834 and GSE28521 was produced from the brain source. Hence, the conclusions generated based on GSE113834 and GSE28521 need to be further validated in the future; (c) ASD is a multifactorial disease, so the investigation of FRGs cannot comprehensively interpret the association of ASD risk.

Collectively, we identified four key FRGs as potential biomarkers and constructed a diagnostic model of childhood ASD, and derived potential criteria for ASD based upon this, that would need future validation. In addition, we found that piperaquine, previously a classic anti-malarial drug, may be worth exploring as a potential therapeutic compound for ASD treatment based on these findings.

## Data Availability Statement

The original contributions presented in the study are included in the article/Supplementary Material, further inquiries can be directed to the corresponding author/s.

## Author Contributions

XW and RL designed the work, integrated and analyzed the data, and wrote the manuscript. QH and XC edited and revised the manuscript. All authors approved the manuscript.

## Conflict of Interest

The authors declare that the research was conducted in the absence of any commercial or financial relationships that could be construed as a potential conflict of interest.

## Publisher’s Note

All claims expressed in this article are solely those of the authors and do not necessarily represent those of their affiliated organizations, or those of the publisher, the editors and the reviewers. Any product that may be evaluated in this article, or claim that may be made by its manufacturer, is not guaranteed or endorsed by the publisher.

## Footnotes

1
http://www.zhounan.org/ferrdb/index.html

2
http://www.pdbbind.org.cn/

3
https://www.drugbank.com/

4
https://www.r-project.org/

## Supplementary Material

Supplementary Figure 1 Venn diagram of key FRGs.

Supplementary Figure 2 The correlation between key FRG expression.
