# Supplementary material for: Development and Validation of a Novel Diagnostic Model for Childhood Autism Spectrum Disorder Based on Ferroptosis-Related Genes
Source: Front Psychiatry. 2022 May 12;13:886055. doi: 10.3389/fpsyt.2022.886055 (PMC9133509; doi:10.3389/fpsyt.2022.886055)
Supplement: Supplementary file 1 [file Table_1.DOCX]

| Table S1. The characteristics of inclusive samples | | | | | | | | |
| --- | --- | --- | --- | --- | --- | --- | --- | --- |
| Characteristics | GSE18123 | | GSE111176 | | GSE113834 | | GSE28521 | |
|  | Normal | ASD | Normal | ASD | Normal | ASD | Normal | ASD |
| No. of subjects | 82 | 41 | 126 | 119 | 12 | 15 | 40 | 39 |
| Age, mean, years | 8.04 | 8.72 | \ | \ | 12.5 | 11.2 | \ | \ |
| Gender, (male/female). N | 48/34 | 31/10 | 126/0 | 119/0 | \ | \ | \ | \ |
| Time of collection of blood samples | \ | \ | \ | \ | \ | \ | \ | \ |
| Postmortem parameters | \ | \ | \ | \ | \ | \ | \ | \ |
| Source | Blood | Blood | Blood | Blood | Brain | Brain | Brain | Brain |

“\” means the data is unavailable.
