# Supplementary material for: Development and Validation of a Novel Diagnostic Model for Childhood Autism Spectrum Disorder Based on Ferroptosis-Related Genes
Source: Front Psychiatry. 2022 May 12;13:886055. doi: 10.3389/fpsyt.2022.886055 (PMC9133509; doi:10.3389/fpsyt.2022.886055)
Supplement: Supplementary file 2 [file Table_2.DOCX]

| **Table S2. Docking score of piperaquine to three FRGs** | | |
| --- | --- | --- |
| **DrugBank_ID** | **Protein** | **Affinity (kcal/mol)** |
| DB13941 (piperaquine) | AKR1C3 | -8.6 |
|  | DDIT4 | -7.7 |
|  | LAMP2 | -7.7 |
