# Supplementary figures and images for: Development and Validation of a Novel Diagnostic Model for Childhood Autism Spectrum Disorder Based on Ferroptosis-Related Genes
Source: Front Psychiatry. 2022 May 12;13:886055. doi: 10.3389/fpsyt.2022.886055 (PMC9133509; doi:10.3389/fpsyt.2022.886055)

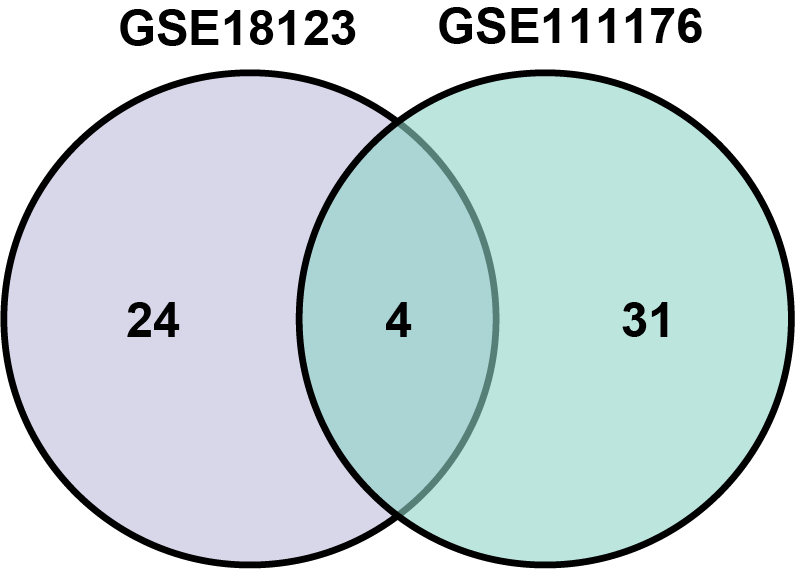

Supplement: Supplementary Figure 1 — Venn diagram of key FRGs. [file Image_1.TIF]

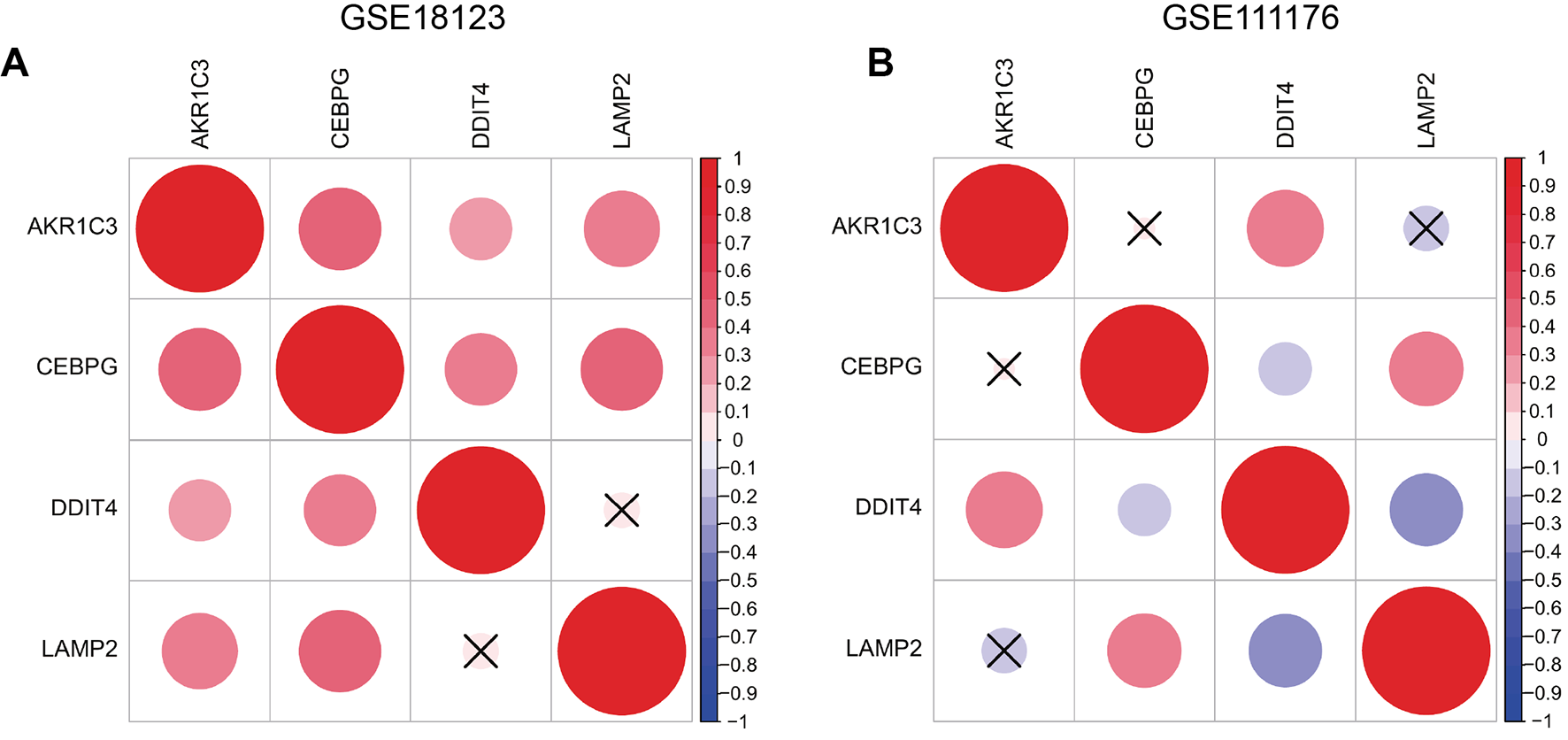

Supplement: Supplementary Figure 2 — The correlation between key FRG expression. [file Image_2.TIF]
